# ﻿A new species of the genus *Serangium* Blackburn (Coleoptera, Coccinellidae) from China, with description of the immature stages

**DOI:** 10.3897/zookeys.1210.129040

**Published:** 2024-08-23

**Authors:** Chu-Yang Huang, Li-Ling Zeng, Wu-wei Fan, Ping Lin, Qi-Jin Dong, Yu-Long Su, Xing-Min Wang

**Affiliations:** 1 College of Plant Protection, South China Agricultural University, Guangzhou 510642, China; 2 Engineering Research Center of Biological Control, Ministry of Education, South China Agricultural University, Guangzhou, 510642, China; 3 Yuxi Branch of Yunnan Provincial Tobacco Company, Yuxi, 653100, China

**Keywords:** Coccinelloidea, larva, Microweiseinae, pupa, taxonomy

## Abstract

A new species *Serangiumxinpingensis* Huang & Wang, **sp. nov.** is described from Yunnan Province, China, as a newly discovered predator on *Bemisiatabaci* Gennadius (Hemiptera, Sternorrhyncha, Aleyrodidae). The new species is a valuable addition to the 14 species of this genus in China known before. A diagnosis, detailed description, including the structure of its immature stages, illustrations, and the distribution of the new species are provided.

## ﻿Introduction

Coccinellidae Latreille, 1807 is the largest family within the superfamily Coccinelloidea, comprises 6896 species recorded worldwide (Wang and Chen 2022). This family has been divided into three subfamilies: Microweiseinae, Monocoryninae and Coccinellinae (Che et al. 2021).

The genus *Serangium* was originally established by Blackburn (1889), with the description of the type species *Serangiummysticum* Blackburn, 1889 from Australia. Chapin (1940) suggested that *Serangium* and similar genera formed a closely related group. Blackwelder (1945) erected for them a separate tribe Serangiini in his checklist, and this was validated by Pope (1962). Currently, the tribe Serangiini is assigned to the subfamily Microweiseinae by Ślipiński (2007). The subfamily Microweiseinae includes four tribes: Carinodulini, Madeirodulini, Serangiini and Microweiseini (Szawaryn et al. 2020).

*Serangium* is the largest genus of the tribe Serangiini, comprising 48 extant species, and mostly occurring in the Oriental and Australian regions (Miyatake 1994; Raimundo and van Harten 2000; Ślipiński and Burckhardt 2006; Wang et al. 2011, 2014). Prior to this study, 14 species of *Serangium* had been known to occur in China (Wang et al. 2011, 2014). In this paper, we described a new species, *Serangiumxinpingensis* sp. nov., from southwestern China. Descriptions of the adult, as well as the fourth instar larva and pupa, are provided.

## ﻿Materials and methods

**Figure 1.**
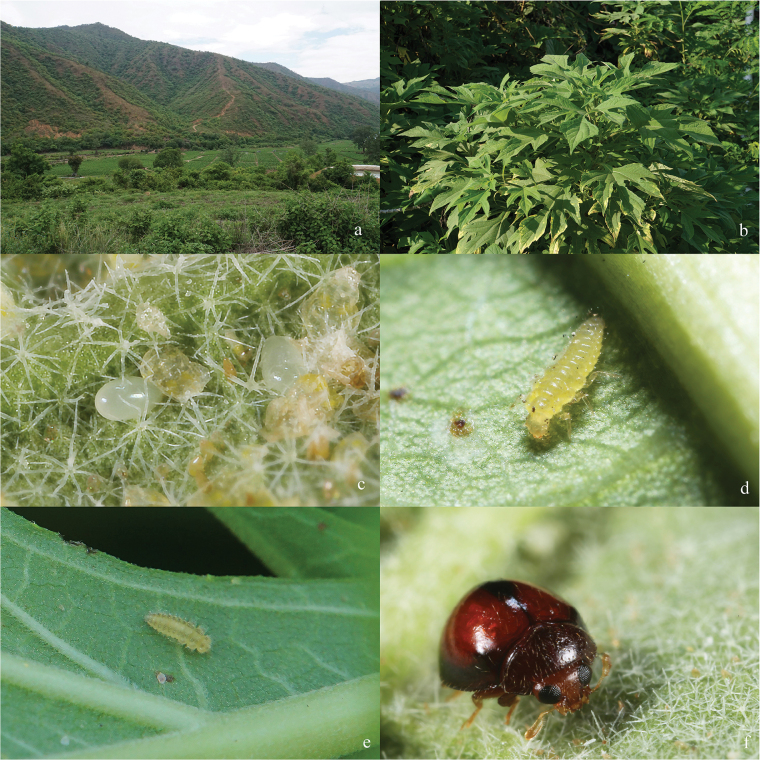
Habitat and ecological photographs of *Serangiumxinpingensis* sp. nov. **a** habitat **b** host plant of *Bemisiatabaci* (Gennadius, 1889) **c** egg **d** second instar larva **e** fourth instar larva **f** adult.

The adult samples examined were collected from Yunnan Province, China. The larval specimens used for this work were obtained by rearing from eggs laid by females of *S.xinpingensis*. We observed this ladybird preying on *Bemisiatabaci* Gennadius infesting *Tithoniadiversifolia* (Hemsl.) A. Gray (Fig. 1). The adults were collected, as well as the plants, and they were raised under laboratory conditions (25 ± 1 °C, 70% ± 10% R.H. and 14:10 h L:D). All examined specimens are deposited in the Department of Entomology of South China Agricultural University, Guangzhou, China (**SCAU**).

The adult morphological terminology used in this paper follows Ślipiński and Tomaszewska (2010), and the larval terminology follows Kamiya (1965) and Gordon and Vandenberg (1991). External structures were observed with a dissecting stereoscope (Zeiss SteREO Discovery V20). The following measurements were made with a micrometer:

**TL** total length, from apical margin of clypeus to apex of elytra;

**TW** total width, across both elytra at widest part;

**TH** total height, through the highest point of elytra to metaventrite;

**HW** head width, including eyes;

**PL** pronotal length, from the middle of the anterior margin to the base of the pronotum;

**PW** pronotal width at widest part;

**EL** elytral length, along the suture, from the apex to the base including the scutellum;

EW = TW.

The examined specimens were stored in 75% ethanol. The specific structures of larvae including mouthparts, head, and tibiotarsus, as well as abdomen of adult, were detached and cleaned with 10% NaOH at 56 C for 1 h. Subsequently, these structures were dissected and rinsed with distilled water. Following processing, the genitalia of both males and females were transferred to neutral balsam, and the specific structures of larvae were transferred to glycerol. Photographs of these structures were taken with an Axiocam 506 color digital camera attached to a Zeiss Image M2 microscope using ZEN 2.3 software. The habitus photographs of larvae and adults were taken with a Canon EOS 5DSR digital camera and processed by using Helicon Focus 7. All photos were processed by using Adobe Photoshop 2023 and Adobe Illustrator 2020.

## ﻿Taxonomy

### ﻿ Serangium

Taxon classificationAnimaliaColeopteraCoccinellidae

Blackburn, 1889

13C104C2-5631-590C-8D02-8F9DD6D44D87

Serangium Blackburn, 1889: 187, 209. Type species, monotypy: Serangiummysticum Blackburn, 1889.
Serangium : Sicard 1909: 150, 151; Chapin 1940: 268; Miyatake 1961: 50; Sasaji 1971: 52; Pang and Mao 1979: 27; Miyatake 1994: 238; Ślipiński and Burckhardt 2006: 39; Ślipiński 2007: 53; Wang et al. 2011: 33.
Semichnoodes Weise, 1892: 15. Type species, monotypy: Semichnoodeskunowi Weise, 1892. Synonymized by Weise 1908: 13.
Catana Chapin, 1940: 266. Type species, original designation, Catanaclauseni Chapin, 1940. Synonymized by Ślipiński and Burckhardt 2006: 39

#### Diagnosis.

**Figure 2.**
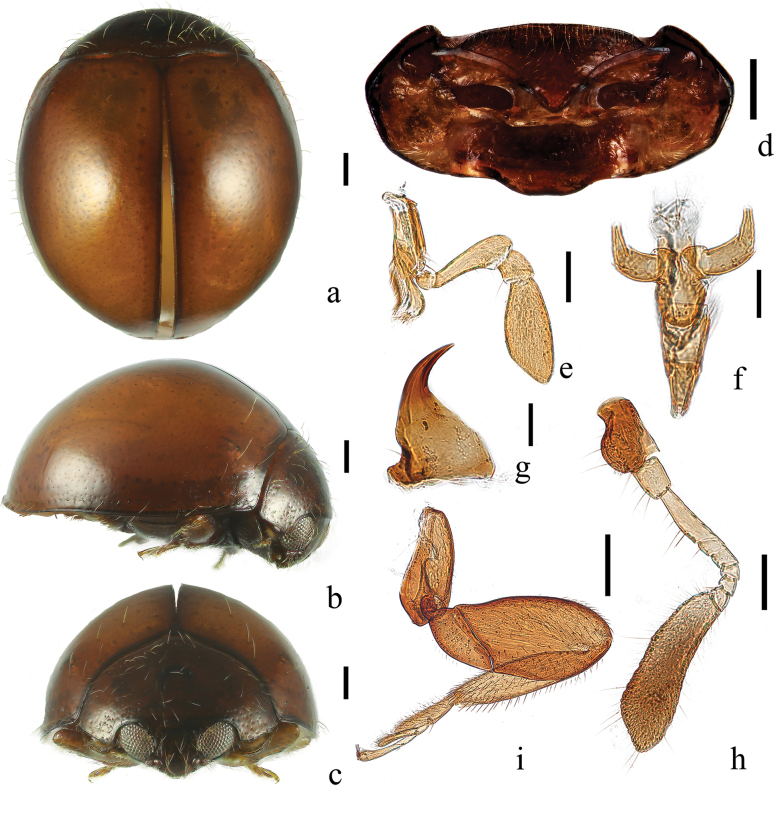
*Serangiumxinpingensis* sp. nov. **a**–**c** habitus photos: **a** dorsal view **b** lateral view **c** front view **d** prothorax, ventral view **e** maxilla, ventral view **f** labium, ventral view **g** mandible, dorsal view **h** antenna, dorsal view **i** posterior leg, ventral view. Scale bars: 0.2 mm (**a–d, i**); 0.1 mm (**e, h**); 0.05 mm (**f, g**).

Body minute, length ranging 1.0–2.5 mm, hemispherical, with head resting closely against prosternal anterior margin at rest; dorsum glossy, bearing sparse, long, thin setae. Mandible small, triangular, with single apical tooth and reduced mola (Fig. 2g); maxillary palps geniculate, palpomeres 2 and 3 closely fitting along stipes, and terminal maxillary palpomere conical or barrel-shaped, always longer than wide (Fig. 2e). Antenna composed of nine antennomeres, antennomere 1 club-shaped and antennomere 3 moderately to strongly elongate (Fig. 2h).

Pronotum short, strongly transverse (Fig. 2d). Scutellum large, triangular. Prosternum strongly protruding medially, forming a broad lobe partly covering mouthparts; prosternal process subtruncate apically, broad. Femora, particularly profemora, broad and flat, closely fitting into depressions on ventral surface and shielding tibiae and tarsi. Elytra smooth, strongly convex, without visible punctures; epipleura extending completely to apex with well-defined cavities accommodating tips of meso- and metafemora (Fig. 2a–c).

**Figure 3.**
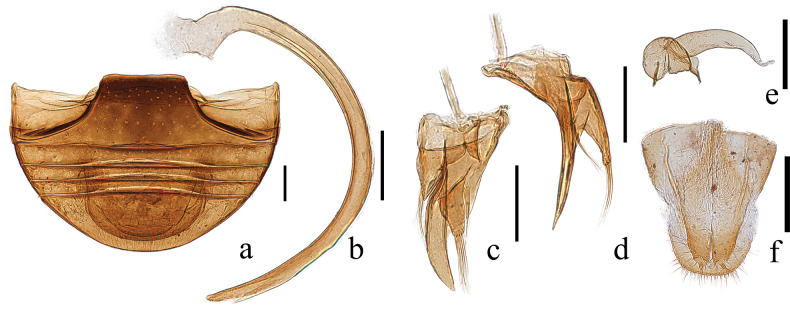
Genitalia of adults of *Serangiumxinpingensis* sp. nov. **a** abdomen, ventral view **b**–**d** male genitalia: **b** penis, lateral view **c** tegmen, ventral view **d** tegmen, lateral view **e**–**f** female ovipositor: **e** spermatheca **f** coxites, ventral view. Scale bars: 0.2 mm.

Abdomen with ventrites 1 and 5 notably longer than ventrites 2–4 together (Fig. 3a). Postcoxal line on abdominal ventrite 1 complete, extending to metanepisternum, without associated pits or pores (Fig. 3a).

### ﻿ Serangium xinpingensis

Taxon classificationAnimaliaColeopteraCoccinellidae

Huang & Wang sp. nov.

FBB17FD1-A1AE-52BF-A203-167D58950EDC

https://zoobank.org/E37802CD-7FFD-40E7-96DD-1084584F0F7D

#### Diagnosis.

This species can be identified by its brown body, dark-brown pronotum with sparse setae and two subparallel longitudinal rows of punctures along the lateral elytral margin, and body integument covered with long, thin setae (Fig. 2a); the penis is strongly arched (Fig. 3b).

This species is very similar to *Serangiumclauseni* (Chapin, 1940) and *Serangiumparcesetosum* Sicard, 1929, but it differs from *S.clauseni* in its more arched penis, squarer bases of parameres, and abdominal ventrite 1 with more densely incised punctures (Wang et al. 2011). It is distinguished from *S.parcesetosum* by its more sharply angular antennomere 2 and curvature of its penis (Booth and Polaszek 1996).

#### Description of adult.

TL: 2.04–2.35 mm, TW: 1.78–2.06 mm, TH: 1.28–1.34 mm, TL/TW: 1.14–1.15; PL/PW: 2.31–3.35; EL/EW: 1.84–1.87.

Body hemispherical; dorsum strongly convex (TH: 1.28–1.34 mm), glabrous (Figs 2a–c). Head brown or reddish-brown. Pronotum brown or reddish-brown, with black margins. Scutellum light brown. Elytra brown, slightly paler than head and pronotum, with one dark-brown band-like marking present along lateral margin; surface with sparse, shallow punctures, demonstrating one row along suture and two subparallel longitudinal rows along lateral margin (Fig. 2a, b).

Head transverse and ventrally flattened, 0.41× elytral width (HW/EW = 2.45). Frons with long sparse setae. Eyes large and coarsely faceted, greatest interocular distance 0.36× of head width. Antenna with nine antennomeres, terminal antennomere large, and spatulately elongate (Fig. 2h).

Pronotum short and strongly transverse, 0.69× of elytral width (PW/EW = 1.96), sparsely covered with long setae. Prosternum with dense setae along anterior margin; prosternal process apically rounded.

**Male genitalia.** Penis strongly curved, arched, gradually narrowing, with blunt tip (Fig. 3b); penis capsule with one small inner process and concave on outer margin (Fig. 3b). Penis guide relatively slender and elongated, wide at base in ventral view, narrowing near distal 1/3 and having tongue-like shape; thin in lateral view, gradually tapering (Fig. 3d). Parameres asymmetrical; one extending from base to approximately 1/3 of penis guide, tapering apically, bearing few long setae; another extending from base to approximately 2/3 of penis guide, wider at base and gradually narrowing apically, with blunt tip, covered with small protuberances and setae (Fig. 3d).

**Female genitalia.** Genital plate (coxites) subtriangular (Fig. 3f), rounded at apex, bearing sparse setae; styli rather long (Fig. 3f), with few setae. Spermatheca consisting of two parts, including one globular, with faint constriction and two small pinch-like projections, and another elongate tubular shaped, slightly tapering distally (Fig. 3e).

#### Description of fourth instar larva.

Length 2.98 mm; width 1.34 mm. Body fusiform, bright yellow overall, dorsal surface with two longitudinal rows of pigmented spots laterally, long setae concentrated on body sides and with sparse, short setae on dorsum.

Head subovoid. Mouthparts light brown; frontal arms U-shaped, distinct. Three bulging stemmata presented on each side at antennal insertions, arranged in triangle (Fig. 5e). Antenna with two antennomeres, antennomere 2 twice as long as antennomere 1, with one long apical sensorium (ca 26.2 μm) and inconspicuous papillae (Fig. 5e). Mandibles heavily sclerotized, subtriangular, without basal tooth, but with one long seta at condyle (Fig. 5g). Maxillae subtriangular (Fig. 5i). Maxillary palp 3-segmented, palpomere 3 nearly subequal in length with palpomere 2, elongate, with sensilla at apex (Fig. 5j), mala transverse and with anterior margin rounded (Fig. 5i). Labrum nearly trapezoidal, with sparse setae on anterior margin. Labium with sparse tomentum and four long setae evenly spaced around apex (Fig. 5f). Labial palps moderately separated, 2-segmented, palpomere 2 distinctly longer than palpomere 1 (Fig. 5j).

Thorax with parallel anterior and posterior margins of each segment, strongly convex on lateral margins; laterally with pigmented spots, bearing long setae. Prothorax narrower than meso- and metathorax, meso- and metathorax almost equal in length and width. Tibiotarsus light brown, elongate, and translucent, with sparse setae; tarsal claws sickle-shaped; basal teeth subtriangular, with one long seta at base (Fig. 5h).

Abdomen 9-segmented, each segment with lateral margin strongly convex, pigmented spots, bearing setae (Fig. 5a, b).

Description of pupa. Length 2.73 mm; width 2.11 mm. Body oval, light yellow, bearing flexible setae.

#### Type materials.

***Holotype***: 1♂, China, Yunnan: Musha Township, Yuxi, 23.8507°N, 101.7782°E, ca 475 m elev., 22.v.2023, Huang CY leg (SCAU). ***Paratypes* (15)**: 9♂♂6♀♀, same data as holotype (SCAU).

#### Distribution.

China (Yunnan).

#### Etymology.

The species epithet “*xinpingensis*” refers to the Xinping County where the type series was collected.

#### Remarks.

**Figure 4.**
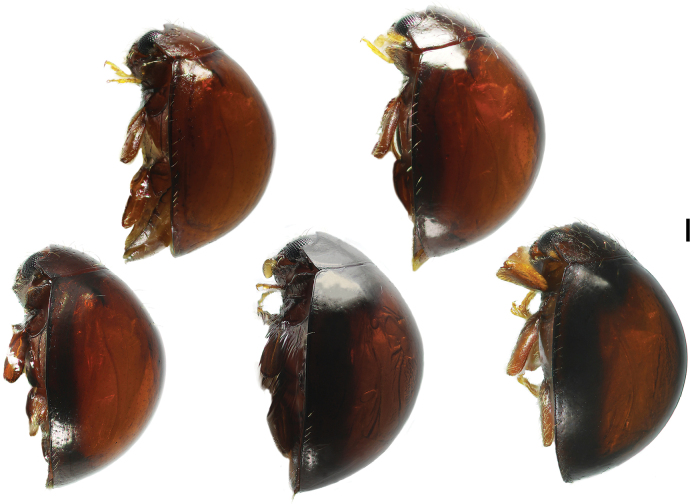
Habitus of adults of *Serangiumxinpingensis* sp. nov., lateral view, showing intraspecific variations of the band-like marking along the outer margin of elytron. Scale bar: 0.2 mm.

This is the first time that individual variation in appearance was found in *Serangium*. Based on our examination of 16 specimens, the male genitalia are highly uniform. The species displays distinct intraspecific variations in the coloration of adults. The head and pronotum coloration frequently subuniform, while the elytra along suture and outer margins may vary from brown to blackish-brown among individuals (Fig. 4).

**Figures 5.**
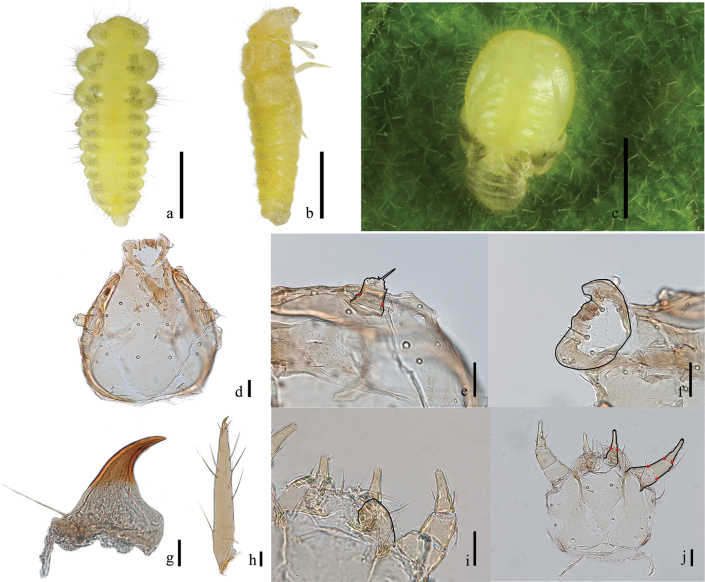
The fourth instar larva and pupa of *Serangiumxinpingensis* sp. nov. **a** fourth instar larva, dorsal view **b** fourth instar larva, lateral view **c** pupa, dorsal view **d**–**j** larval structures: **d** head capsule, dorsal view **e** antenna, dorsal view **f** labrum. dorsal view **g** mandible, dorsal view **h** tibiotarsus and claw. dorsal view **i** maxillary mala, ventral view **j** mouthparts, ventral view. Scale bars: 1 mm (**a–c**); 0.05 mm (**d–f, h–j**); 0.025 mm (**g**).

Larvae of the subfamily Microweiseinae have been given little attention, and only two species have been recorded so far, namely *Scymnomorphusjaponicus* (Kamiya, 1960) and *Serangiumjaponicus* Chapin, 1940, belonging to the tribes Microweiseini and Serangiini, respectively. The larva of *Scymnomorphusjaponicus* was described by Kamiya (1965) and Sasaji (1968), although it was originally described under the name *Sukunahikonajaponicus* Kamiya, 1960. The known larvae of the two tribes Serangiini and Microweiseini are very similar, but they differ greatly in the antennomere 2, which is much shorter in the tribe Microweiseini than Serangiini (Kamiya 1965). Within the tribe Serangiini, the larva of the new species can be distinguished from *S.japonicus* by its distinct U-shaped frontal arms. In *S.japonicus*, the frontal arms are invisible (Jiaming Zhuang pers. obs.)

## Supplementary Material

XML Treatment for Serangium

XML Treatment for Serangium xinpingensis

## ﻿Additional information

### Author ORCIDs

Chu-Yang Huang https://orcid.org/0000-0001-5943-7088

Li-Ling Zeng https://orcid.org/0009-0007-9858-0638

### Data availability

All of the data that support the findings of this study are available in the main text or Supplementary Information.
